# Advancing precision tacrolimus therapy: a systems genetics dissection in BXD platform

**DOI:** 10.3389/fimmu.2026.1903968

**Published:** 2026-08-26

**Authors:** Wei Zhou, Xin-Bo Wang, Yu-Wei Guo, Bing Hu, Zi-Xuan Wang, Yu Xiong, Xuan-Ming Chen, Bo-Yang Xu, Xiao-Xing Wang, Xiao-Xue Wang, Fei-Fei Han, Jun-Jie Wang, Hao-Jun Zhang, Liang Peng, Li-Li Gong, Lu Lu, Li-Hong Liu

**Affiliations:** 1 Department of Pharmacy, China-Japan Friendship Hospital, Beijing, China; 2 China-Japan Friendship Hospital (Institute of Clinical Medical Sciences), Chinese Academy of Medical Sciences & Peking Union Medical College, Beijing, China; 3 Department of Pharmacy, Aerospace Center Hospital, Peking University Aerospace School of Clinical Medicine, Beijing, China; 4 Maurice H. Kornberg School of Dentistry, Temple University., Philadelphia, PA, United States; 5 Institute of Clinical Medical Sciences, China-Japan Friendship Hospital, Capital Medical University, Beijing, China; 6 Department of Pharmacy, Mianyang Central Hospital, School of Medicine, University of Electronic Science and Technology of China, Mianyang, China; 7 Institute of Medicinal Biotechnology, Chinese Academy of Medical Sciences & Peking Union Medical College, Beijing, China; 8 China-Japan Friendship Hospital, Beijing, China; 9 Beijing Chao-Yang Hospital, Capital Medical University, Beijing, China; 10 Department of Genetics, Genomics and Informatics, University of Tennessee Health Science Center, Memphis, TN, United States

**Keywords:** CYP2A6, DBP, precision medicine, tacrolimus, The BXD recombinant inbred mouse strains

## Abstract

**Background:**

Tacrolimus is a core immunosuppressant in organ transplantation, but its narrow therapeutic window and significant pharmacokinetic variability hinder precision dosing. Although CYP3A5-guided strategies have established clinical relevance for tacrolimus initial dose adjustment, they do not fully account for the marked interindividual variability in tacrolimus exposure, highlighting the need for complementary models to decode more complex genetic regulation. This study aimed to identify candidate genetic modulators of tacrolimus metabolism and develop an integrated predictive framework for individualized therapy.

**Methods:**

Using 46 BXD recombinant inbred mouse strains, we characterized transcriptomics and machine learning, and validated key genes. We then constructed a clinical model using data from 168 renal transplant recipients.

**Results:**

We identified 19 genomic loci associated with tacrolimus pharmacokinetic traits and supported DBP/CYP2A6 as candidate modulators associated with tacrolimus disposition. The clinical prediction model, incorporating these genes and clinical variables, achieved robust AUROC.

**Conclusions:**

These findings support a polygenic contribution to tacrolimus metabolism and provide an experimental and computational framework for identifying candidate modulators relevant to individualized dosing. The BXD mouse platform offers a systems-genetics approach for mechanistic discovery that may inform future translational studies on tacrolimus precision dosing.

## 1 Background

Tacrolimus (FK506) is a cornerstone immunosuppressant in organ transplantation, preventing allograft rejection through calcineurin inhibition and subsequent suppression of interleukin-2 (IL-2) driven T-cell activation (1, 2). Despite its established efficacy, tacrolimus exhibits a narrow therapeutic window, with dosing inaccuracies posing particularly high risks during the early post-transplant phase (3–5). Even minor deviations in drug exposure during this critical window can precipitate irreversible graft loss (6, 7). Although therapeutic drug monitoring (TDM) is routinely used, its retrospective nature results in a clinically significant time lag, limiting its utility for timely dose optimization (8, 9). Consequently, clinicians continue to rely on empirical weight-based dosing at therapy initiation, often accepting the necessity of multiple subsequent dose adjustments (10).

Tacrolimus dosing is further complicated by substantial interindividual pharmacokinetic variability, influenced by both genetic and non-genetic factors (7). Although CYP3A5 is the most established pharmacogenetic marker for tacrolimus initial dose adjustment and has recognized clinical relevance, it explains only part of the observed interindividual variability in tacrolimus exposure (11–16). Beyond CYP3A5, polymorphisms in genes including CYP3A4, ABCB1, POR, and IL-10 exert only modest effects on tacrolimus pharmacokinetics, and their clinical utility is constrained by inconsistent inter-population findings (12, 17, 18). The clinical constraint of genotype-guided dosing is further evidenced by the 5-year follow-up data from the French TACTIQUE study (2016), in which genotype-based dosing improved early target attainment but failed to enhance long-term outcomes such as graft survival or rejection rates (19). Adding to this complexity are several dynamic clinical variables, including age, liver and kidney function, metabolic status, and drug–drug interactions, all of which contribute to the intricate pharmacokinetic network governing tacrolimus exposure (20, 21). Together, these findings highlight the need for integrated and complementary strategies that extend beyond single-gene approaches while remaining grounded in established pharmacogenetic knowledge. Current precision dosing research relies primarily on two approaches: large human cohort studies and conventional animal models, each with significant limitations. While cohort studies reflect real-world clinical variability, they are often costly and time-intensive. Conversely, standard laboratory animal models such as C57BL/6J, with their homogeneous genetic backgrounds, fail to recapitulate the phenotypic diversity of human populations, resulting in limited clinical translatability. There is thus a clear need for novel model systems that integrate genetic diversity with experimental controllability. Genetic Reference Panels (GRPs) provide a powerful alternative, offering defined genetic structures, reproducible environments, and high experimental consistency (22). This study utilized the BXD recombinant inbred panel, a community resource of over 150 strains derived from C57BL/6J and DBA/2J founders, whose high-resolution genomes have been fully sequenced, providing a powerful platform for mapping complex traits and identifying therapeutic targets (23). Through the BXD platform, a coherent workflow from phenotype mapping to gene discovery and dosing guidance has been established. This strategy has already proven effective in identifying key genetic regulators—such as Ndufs2 and Scp2—in studies of response variability to tetrahydrocannabinol and nicotine, demonstrating the broad utility of GRPs in advancing precision dosing research (24, 25). However, existing applications of this strategy have been limited by several factors: the relatively small number of strains analyzed in most studies restricts statistical power and genetic resolution; furthermore, many previous studies relied solely on QTL mapping without functionally validating candidate genes or confirming findings in human cohorts. Our current study was specifically designed to bridge these gaps by implementing an integrative strategy that combines multi-omics profiling with experimental and clinical validation.

In this study, to investigate the precision medicine of tacrolimus using the BXD panel, we utilized a QTL mapping approach to identify genomic regions associated with variability in tacrolimus pharmacokinetics and pharmacodynamics across the BXDs. QTL analysis is a powerful tool in genetic research, enabling the localization of genes that contribute to complex traits by correlating phenotypic variation with genetic markers across the genome (26). By integrating these phenotypic data with high-resolution genetic maps, we aim to pinpoint key genetic determinants contributing to tacrolimus response variability, followed by experimental validation using both *in vitro* and *in vivo* models. Complementing this genetic analysis, we incorporated clinical data from a retrospective cohort of renal transplant recipients. By merging the QTL-derived genetic markers with clinically measured tacrolimus exposure data, we developed a machine learning‐based model to estimate individualized drug exposure, offering a scalable strategy for precision immunosuppression.

## 2 Methods

### 2.1 Materials

Tacrolimus (purity ≥ 98%) was purchased from Dalian Meilun Biotechnology Co., Ltd. (Dalian, China). Meanwhile, pharmaceutical-grade olive oil was obtained from Shanghai McLean Biochemical Technology Co., Ltd. (Shanghai, China). Low-passage 293T cells were purchased from Beyotime Biotechnology Co., Ltd. (Shanghai, China). High-glucose Dulbecco’s Modified Eagle Medium (DMEM) was sourced from Thermo Fisher Scientific. Additionally, HPLC-grade acetonitrile and glacial acetic acid (100%) were purchased from Thermo Fisher Scientific, while ultra-pure deionized water (18.2 MΩ cm) was obtained from Hangzhou Wahaha Co., Ltd. (Hangzhou, China). The construction of adeno-associated virus (AAV) vectors and viral packaging for m-Cyp2a22 and m-Dbp were purchased from Hanheng Biotechnology. Dual Luciferase Reporter Assay Kit (DL101-01) was purchased from Vazyme Biotech Co.,Ltd.

### 2.2 Animal

A total of 414 male mice across 46 BXD strains were bred in-house at China-Japan Friendship Hospital. These strains, imported by the hospital from the University of Tennessee (USA) in 2022, have established the institution as the only one in China currently authorized to utilize BXD mice. Prior to the experiment, all mice were housed under standard specific pathogen-free (SPF) conditions: controlled lighting (12-h light/12-h dark cycle), proper ventilation, temperature maintained at 25 ± 2°C, and relative humidity set at 60%.

### 2.3 Pharmacokinetics

Forty-six distinct BXD mouse strains served as the study subjects, with 9 male mice (20 ± 2g) included per strain. Prior to the experiment, the mice were fasted for 12 hours, with ad libitum access to water. Pharmaceutical-grade olive oil was used to prepare a tacrolimus suspension at a concentration of 1mg/mL. After a single oral gavage administration at a dose of 10 mg/kg, which was selected for pharmacokinetic profiling across the BXD panel, 80 µL of whole blood was collected via orbital venous plexus puncture at the following time points: 5, 15, and 30 minutes, as well as 1 h, 2 h, 4 h, 6 h, 8 h, 12 h and 24 hours post-administration (27). The collected whole blood was transferred to pre-chilled blood collection tubes containing EDTA, thoroughly mixed, and stored at -80 °C until subsequent analysis.

### 2.4 Sample preparation

150 μL blank acetonitrile was added to 50 μL blood sample. The mixture was swirled by a vortex for 0.5 min and then centrifuged twice at 14,000 RPM (Revolutions Per minute) for 10 min 2 μL of supernatant were analyzed by LC-MS/MS method.

### 2.5 Instrumentation and analytical conditions for quantification analysis

LC-MS/MS system is composed of Q Exactive Orbitrap mass and Thermo Vanquish liquid chromatograph (Thermo Scientific, USA) (28). Zorbax-SB C18 column (2.1 ×50 mm, 1.7 µm Agilent USA) was used to separate the analytes. The mobile phase consisted of water containing 0.1% formic acid (Phase A) and acetonitrile (Phase B) at a flow rate of 0.25 mL/min with an operating temperature at 40 °C. The entire elution system was set to last 4.5 min with a gradient elution system: 0.00 to 0.60 min, 40% to 100% B; 0.60 to 2.50 min, 100% B; 2.50 to 2.51 min, 100% to 40% B; 2.51 to 4.50 min, 40% B. The automatic sampling chamber was maintained at 8 °C throughout the analysis. Positive ion scanning mode in Parallel Reaction Monitor (PRM) mode was used for mass spectrometry analysis. The optimized ionspray voltage and temperature were 3500 V and 320 °C. Nitrogen was used as sheath gas at 35 psi and auxiliary gas at 7 psi. The collision energy (CE) of tacrolimus was 16 Ev. Quantification was performed using the PRM transitions of m/z 821.5158 → 840.717 for tacrolimus.

### 2.6 AFADESI-MSI analysis

Target tissues were harvested after animals were sacrificed by decapitation under anesthesia, then stored at -80 °C until sectioning into 15 µm slices using a CM 1860 UV cryostat microtome. Sections were thaw-mounted onto slides, stored in sealed containers at -80 °C, and vacuum-dried for 60 min before AFADESI-MSI analysis. The AFADESI-MSI platform was built by replacing the original ion source of a Q Exactive mass spectrometer (Thermo Scientific, Bremen, Germany) with a custom AFADESI ion source. High-velocity airflow enhanced ion formation/collection; a 500 mm stainless steel tube (OD 4 mm, ID 3 mm) facilitated ion transport. An XY stage — controlled by custom software and an SC100 stepper motor (Beijing Optical Century, Beijing, China)-regulated movement beneath the ion source. AFADESI-MSI was performed in positive/negative ion modes on the Q Exactive over m/z 70-1000. Tissue sections on a 3D stage moved in 0.2 mm steps (horizontal/vertical). Spray solvent was acetonitrile/water (8:2, v/v) at 5 µL/min. Spatial resolution was around 100 µm; airflow and capillary temperature were set to 40 L/min and 350°C respectively.

### 2.7 RT-qPCR

Total RNA was isolated from mouse liver or intestinal tissues using TRIzol reagent (Takara, Japan). cDNA synthesis was performed with HiScript II Reverse Transcriptase (Vazyme, Cat# R323). Real-time quantitative PCR (qPCR) was conducted on the QuantStudio 5 system (Thermo Fisher ABI) using SYBR Green PCR Master Mix (Vazyme, Cat# Q712) to determine the expression levels of target genes. β-actin served as the reference gene. The sequences of RT-qPCR primers are provided in **Supplementary Table 1**.

### 2.8 Quantification and statistical analysis

QTL mapping-In order to investigate the influence of genetic backgrounds among different BXD strains on tacrolimus pharmacokinetics, we employed quantitative trait loci (QTL) analysis for our investigation. Specifically, the R/qtl package was utilized to conduct QTL mapping for PK parameters, encompassing are AUC_0-t_, T_max_, C_max_, MRT_0-t_, and T_1/2_ (26). A Haley–Knott regression (hk) method was applied for QTL scanning, and the significance of QTLs was determined through 10,000 permutation tests. Genome-wide significant loci were defined as those exceeding the trait-specific LOD threshold corresponding to permutation p < 0.05. Suggestive loci were defined using the conventional suggestive threshold corresponding to one expected false positive per genome scan, following Lander and Kruglyak (29).

### 2.9 Transcriptomic analysis and gene identification

BXD mice from 11 strains at the extremes of the distribution were assigned to High-AUC_0-t_ (n = 17) and Low-AUC_0-t_ (n = 13) groups. Immediately after the 24 h bleed, liver, kidney and jejunal segments were excised, snap-frozen, and total RNA extracted. Strand-specific libraries were constructed and sequenced on an Illumina NovaSeq 6000 (150 bp paired-end). Reads were aligned to GRCm39 with STAR v2.7.9a, and differential expression analysis was performed with DESeq2. Functional enrichment (GO and KEGG) was carried out using clusterProfiler v4.0 with Benjamini-Hochberg correction (*p* < 0.05). Gene-set enrichment analysis (GSEA) employed the fgsea package against the MsigDB v7.5 C2 canonical pathway collection. Previously defined tacrolimus QTLs (LOD ≥ 3.0) were intersected with differentially expressed genes via bedtools v2.30 to yield candidate quantitative trait genes (QTGs).

### 2.10 Machine learning-based feature gene selection

Samples were randomly split into training (70%) and test (30%) sets while maintaining the original class balance. Four algorithms were executed exactly as implemented in the analysis pipeline: (i) elastic-net logistic regression (Enet α = 0.9, 10-fold CV, λmin); (ii) LASSO logistic regression (α = 1, 10-fold CV, λmin); (iii) stepwise logistic regression (glm, family = binomial, bidirectional AIC-based selection, trace = 0); and (iv) SVM with radial basis kernel (caret::rfe, svmRadial, 10-fold CV, functions = caretFuncs). Genes retained by each algorithm were recorded and intersected; those present in at least two of the four lists were defined as consensus feature genes. Variable importance of the consensus set was subsequently assessed with a random forest model (randomForest, ntree = 500, mtry = √p) using the mean decrease in Gini impurity.

### 2.11 Experiment on construction and packaging of adeno-associated virus vectors

Adeno-associated viruses (AAVs) carrying m-Cyp2a22 and m-Dbp were respectively administered via tail vein injection to BXD139 and BXD170 mouse strains at a titer of 1×10^12^ TU/mL. Three weeks later, the mice received a single oral gavage of tacrolimus at a dose of 10 mg/kg. Subsequent blood collection was performed via orbital venous plexus puncture at the following time points: 5, 15, 30 min, 1, 2, 4, 6, 8, 12 and 24 h post-administration. The collected blood samples were stored at -80 °C until subsequent analysis.

### 2.12 Luciferase reporter assay

Luciferase reporter vectors harboring the promoters of CYP3A4 and CYP3A5 were constructed by Shanghai GeneChem Co., Ltd. For the subsequent experiments, 293T cells were employed, and each of the two reporter vectors was co-transfected with the internal reference vector pRL-TK plus either the Dbp overexpression vector pcDNA3.1-Dbp (designated as the experimental group) or the empty vector pcDNA3.1-empty (designated as the control group), respectively. At 48 hours post-transfection, the cells were lysed, and the activities of firefly luciferase and Renilla luciferase were assayed. Based on the detected activities, the relative luciferase activity was calculated.

### 2.13 sample collection and genomic profiling

Residual EDTA-anticoagulated whole-blood samples were collected between 2018 and 2019 from 168 renal-transplant recipients receiving tacrolimus-based immunosuppressive therapy.

Demographic data, tacrolimus trough concentrations, daily doses and routine biochemical parameters were extracted from electronic medical records. Samples were anonymised, bar-coded and shipped on dry ice to CWBiO Biotechnology Co., Ltd. (Taizhou, China). Genomic DNA was extracted and libraries constructed according to the protocol; sequencing was performed on a PromethION 2 Solo instrument using R10.4.1 flowcells (ONT FLO-PRO114M). FASTQ files were quality-filtered and adapter-trimmed with Fastplong v0.2.2. Clean reads were aligned to the GRCh38 reference with Minimap2 v2.28-r1209, and variant calling was performed. PLINK v1.9 was used to test for associations between genotype and tacrolimus exposure (high vs. low trough concentration groups) under an additive model. Variants with p < 0.05 were retained as nominally associated candidates for downstream feature selection; this threshold was applied as a dimensionality-reduction pre-filtering step prior to machine learning rather than as a formal test of statistical significance.

### 2.14 Construction of the tacrolimus prediction model

A composite dataset integrating germline variant calls, first tacrolimus dose (mg), serum creatinine (µmol L^-^¹), ALT (U L^-^¹) and AST (U L^-^¹) was assembled. Because these variables differed substantially in scale, they were normalized before model fitting. Missing values were present were encoded as 0 in the normalized clinical-variable matrix, corresponding to mean-level imputation on the normalized scale. Eight machine-learning algorithms—Enet, gradient-boosting machine (GBM), GLM, J48-based classifier (JCS), RF, Ridge regression, SVM and LASSO—were applied independently to rank input features. Variables selected by at least five of the eight algorithms were retained as consensus predictors. Using these consensus features, seven modelling strategies (Enet, LASSO, rpart, glm, partial least-squares generalized linear model (plsRglm), RF and SVM) were trained on 70% of the data and validated on the remaining 30%, maintaining class balance. Model performance was evaluated by receiver-operating characteristic (ROC) curves generated with the pROC package; area under the ROC curve (AUROC) was reported for the training set, test set and the entire cohort. To provide an unbiased estimate of model performance and address potential feature-space leakage introduced by the upstream PLINK pre-filtering step, we implemented 5-fold nested cross-validation. In each outer fold, the outer test partition was held out entirely; all LASSO feature selection and hyperparameter tuning (10-fold inner CV, α = 1, λ = λmin) were performed exclusively within the outer training partition. Model discrimination was evaluated by AUROC for each outer test fold, and results are reported as mean ± SD across five folds. A parallel clinical-variables-only model (creatinine, ALT, AST, and tacrolimus dose) was constructed using the same nested CV framework to assess the incremental predictive contribution of the genetic variables. Model calibration was further assessed using calibration plots based on out-of-fold predicted probabilities from the nested cross-validation framework. Calibration performance was summarized using the Brier score, calibration intercept, and calibration slope.

### 2.15 Statistical analysis

All experimental data are presented as mean ± standard error of the mean (SEM). WinNonlin software (version 8.0) was used to fit plasma concentration data and calculate pharmacokinetic parameters via non-compartmental analysis.

## 3 Results

### 3.1 Pharmacokinetic Study of Tacrolimus in 46 Strains of BXD Mice

**Figure 1:**
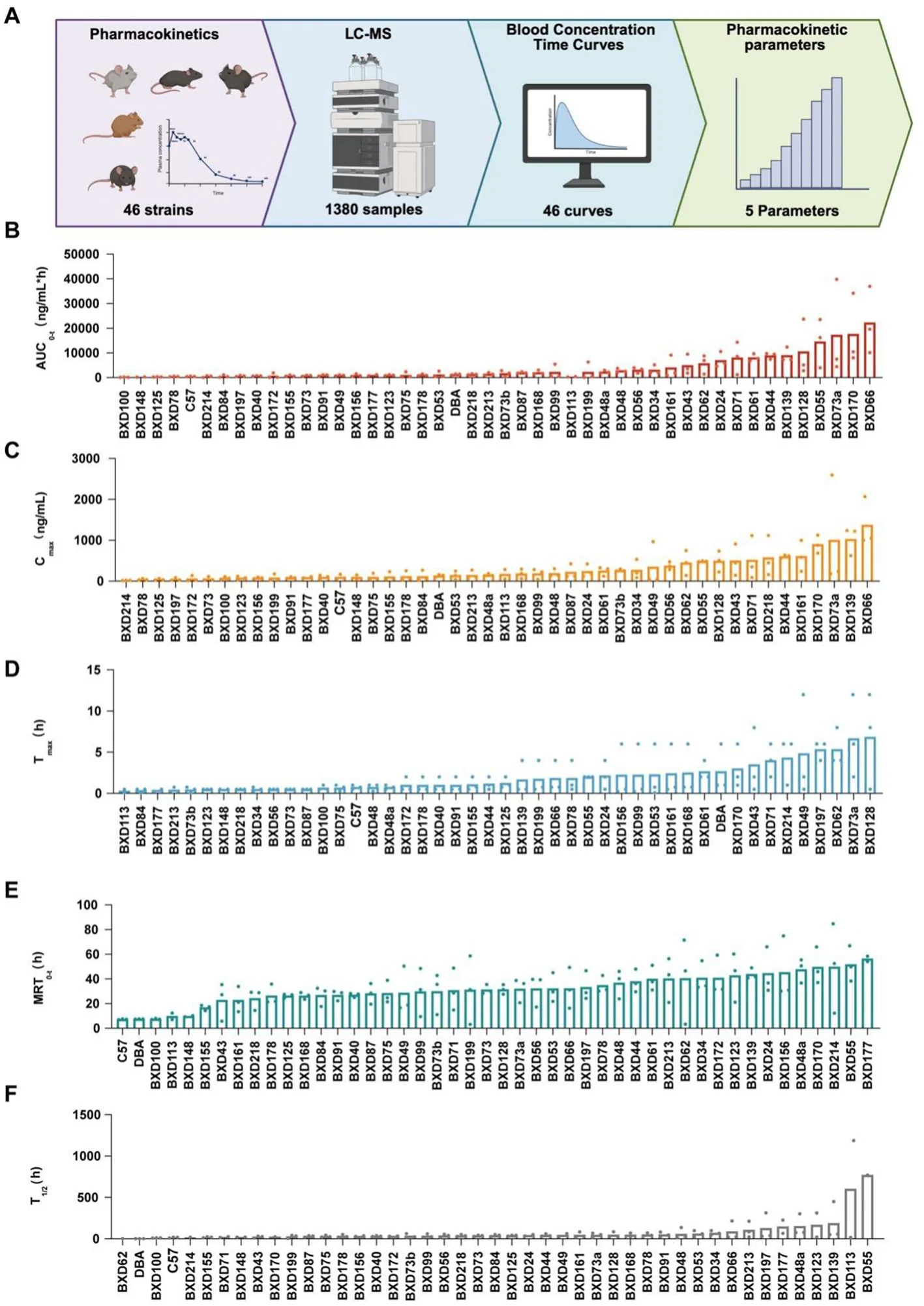
Pharmacokinetic study of tacrolimus in 46 strains of BXD mice. **(A)** Workflow of Pharmacokinetic Research of Tacrolimus in 46 Strains of BXD Mice. **(B)** AUC_0-t_ of Tacrolimus in 46 Strains of BXD Mice. **(C)** C_max_ of Tacrolimus in 46 Strains of BXD Mice. **(D)** T_max_ of Tacrolimus in 46 Strains of BXD Mice. **(E)** T_1/2_ of Tacrolimus in 46 Strains of BXD Mice. **(F)** MRT_0-t_ of Tacrolimus in 46 Strains of BXD Mice.

This study systematically characterized the pharmacokinetic properties of tacrolimus across 46 distinct BXD mouse strains. The blood concentrations of the drug were quantified via liquid chromatography-mass spectrometry (LC-MS), with a total of 1380 biological samples collected. These samples were subsequently used to construct 46 blood drug concentration-time curves and derive five key pharmacokinetic parameters (**Figure 1A**; **Supplementary Figure 1** Supporting Information).

Non-compartmental analysis (NCA) was performed to calculate the aforementioned key pharmacokinetic parameters, including time to peak concentration (T_max_), peak concentration (C_max_), total exposure (AUC_0-t_), half-life (T_1/2_), and mean residence time (MRT_0-t_) (**Figures 1B–F**). Notably, the pharmacokinetic profile of tacrolimus in BXD mice exhibited substantial inter-strain variability. This observation not only established a robust foundation for investigating the genetic regulatory mechanisms underlying tacrolimus pharmacokinetics but also provided phenotypic data to inform subsequent gene identification efforts.

### 3.2 Analysis of tacrolimus concentrations in various organs and tissues of special BXD mouse strains via AFADESI-MSI

**Figure 2:**
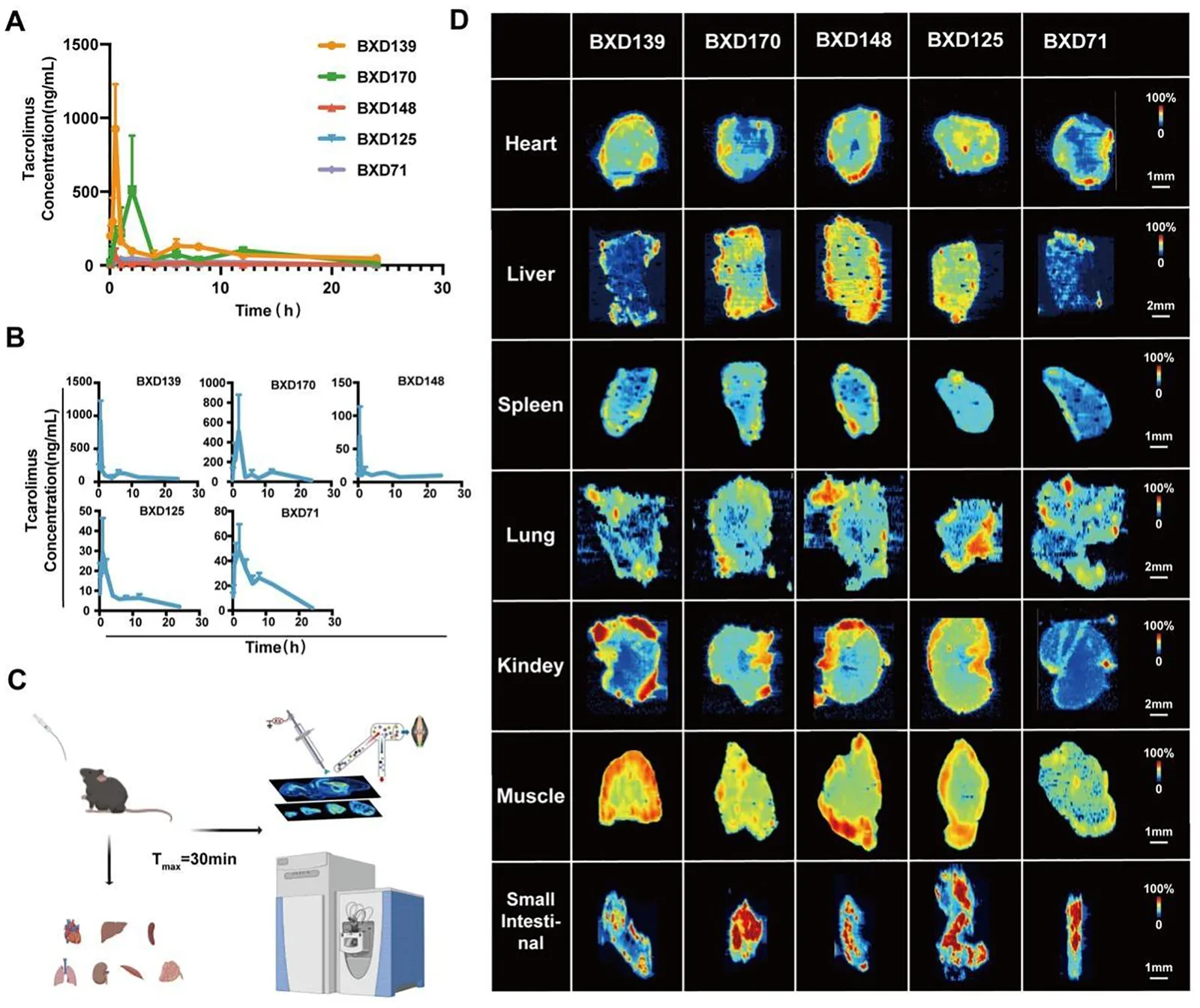
Study on tacrolimus concentration differences in various tissues and organs of special BXD strain mice using MSI. **(A, B)** Mean Tacrolimus Blood Concentration-Time Curves in BXD139, BXD170, BXD148, BXD125 and BXD71 Mouse Strains (60 mg/kg, Oral Administration). **(C)** Schematic illustration of the mass spectrometry imaging strategy. **(D)** Spatial Metabolic Imaging of Tacrolimus in Various Organs (Heart, Liver, Spleen, Lung, Kidney, Muscle, Small Intestine) of Different BXD Mouse Strains.

Based on the pharmacokinetic results obtained from BXD mice, we selected 5 strains with significant differences in blood drug concentration, namely BXD139, BXD170, BXD148, BXD125 and BXD71 (**Figures 2A, B**). Among these strains, BXD139 and BXD170 exhibited relatively high AUC_0-t_ values, while BXD125, BXD71, and BXD148 showed relatively low AUC_0-t_ values. AFADESI-MSI analysis revealed that 30 minutes after oral administration of tacrolimus (at a dose of 60 mg/kg), significant inter-strain differences in tacrolimus concentration were observed across various organs and tissues (**Figures 2C, D**; **Supplementary Figure 2**). A higher dose (60 mg/kg) was intentionally used in the AFADESI-MSI experiments to improve tissue-level detection sensitivity. Notably, tacrolimus exhibited distinct tissue distribution patterns across the BXD strains, with the highest accumulation generally observed in the small intestine. Organ-specific analysis revealed elevated drug concentrations in the heart of BXD125, BXD139, and BXD148, in contrast to the lower levels observed in BXD170 and BXD71. In the liver, higher levels were detected in BXD125, BXD170, and BXD148, while lower levels were found in BXD139 and BXD71. In the spleen, BXD148 showed uniquely high tacrolimus accumulation, whereas the other four strains exhibited relatively low concentrations. Pulmonary accumulation was prominent in BXD125 and BXD148 but reduced in BXD139, BXD170, and BXD71. Renal concentrations were markedly elevated in BXD139 compared to the other strains. Similarly, muscle tissue displayed higher drug levels in BXD139 and BXD148, while BXD125, BXD170, and BXD71 showed limited accumulation. In conclusion, among the BXD strains with significant differences in blood tacrolimus concentration, notable variations in its concentration were also observed across different organs or tissues.

### 3.3 Genetic contributors of tacrolimus metabolic variation

**Figure 3:**
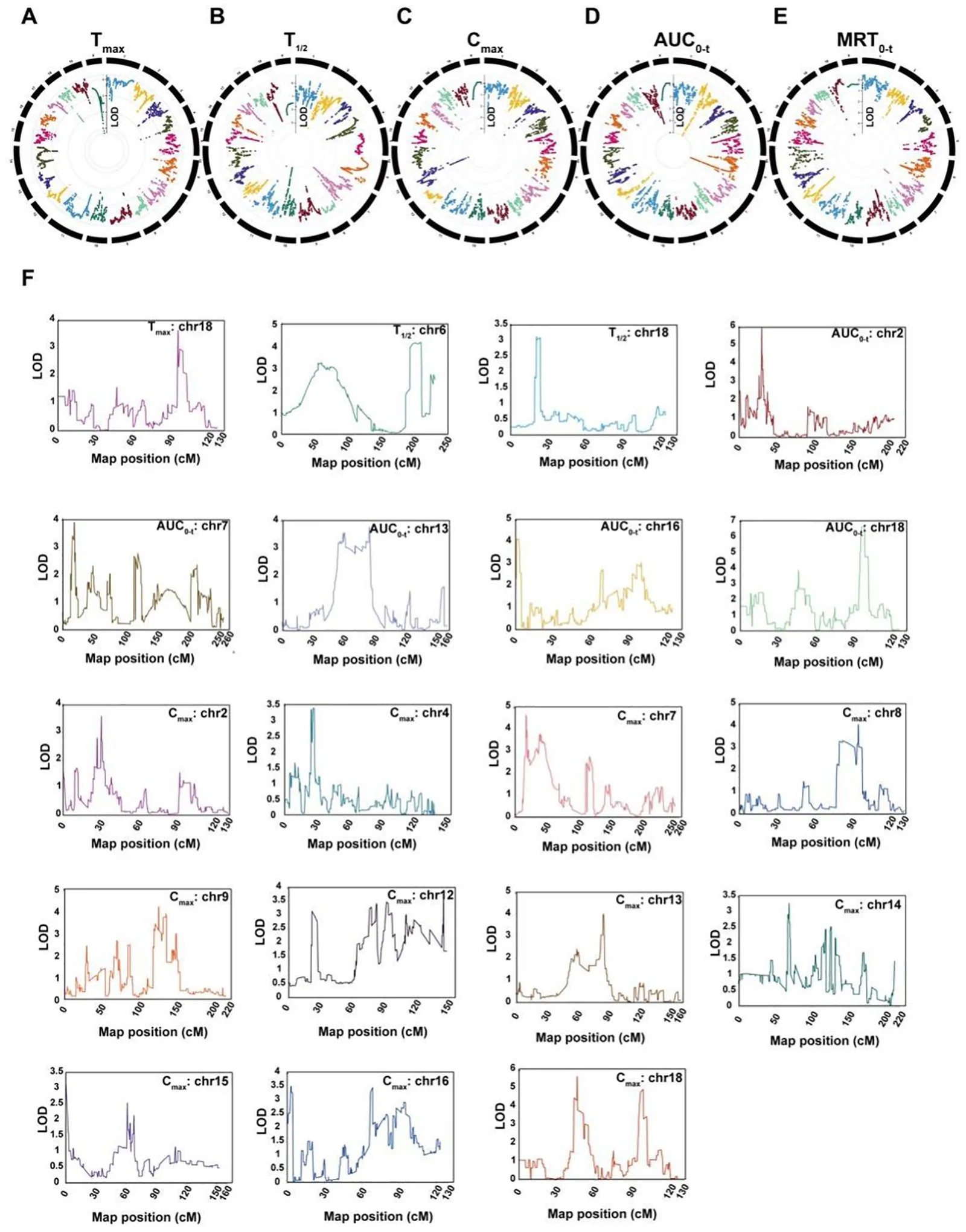
Genome-wide QTL mapping of tacrolimus pharmacokinetic parameters in the BXD cohort (n = 46 strains; Haley-Knott regression with 10,000 permutations). **(A)** QTL mapping of AUC_0-t_. **(B)** QTL mapping of C_max_. **(C)** QTL mapping of T_1/2_. **(D)** QTL mapping of T_max_. **(E)** QTL mapping of MRT_0-t_. **(F)** Composite Manhattan plot highlighting chromosomes (Chr) harboring QTLs with LOD > 3 across all parameters. Genome-wide significance (*p* < 0.05).

We observed significant inter-strain variability in tacrolimus metabolism across 46 BXD mouse strains. QTL mapping was employed to investigate the genetic underpinnings of these metabolic differences. Genome-wide QTL analysis was conducted on the pharmacokinetic parameters, including T_max_, T_1/2_, C_max_, AUC_0-t_, and MRT_0-t_. (**Figures 3A–E**; **Supplementary Figure 3**). This approach identified 19 QTL loci associated with tacrolimus pharmacokinetics, comprising 4 genome-wide significant loci (AUC_0-t_: Chr2 LOD = 5.89, Chr18 LOD = 6.61; C_max_: Chr7 LOD = 4.62, Chr18 LOD = 5.61) and 15 suggestive loci across four pharmacokinetic traits (**Figure 3F**; **Supplementary Table 2**).

Additionally, QTL analysis of blood concentrations at multiple timepoints across strains revealed that the implicated loci substantially overlapped with those regulating the core PK parameters. This pattern is consistent with a shared genetic contribution to tacrolimus variation across BXD strains and supports these loci as candidate modulators of tacrolimus disposition.

### 3.4 Transcriptomic dissection reveals drug-metabolism divergence between extreme BXD strains

**Figure 4:**
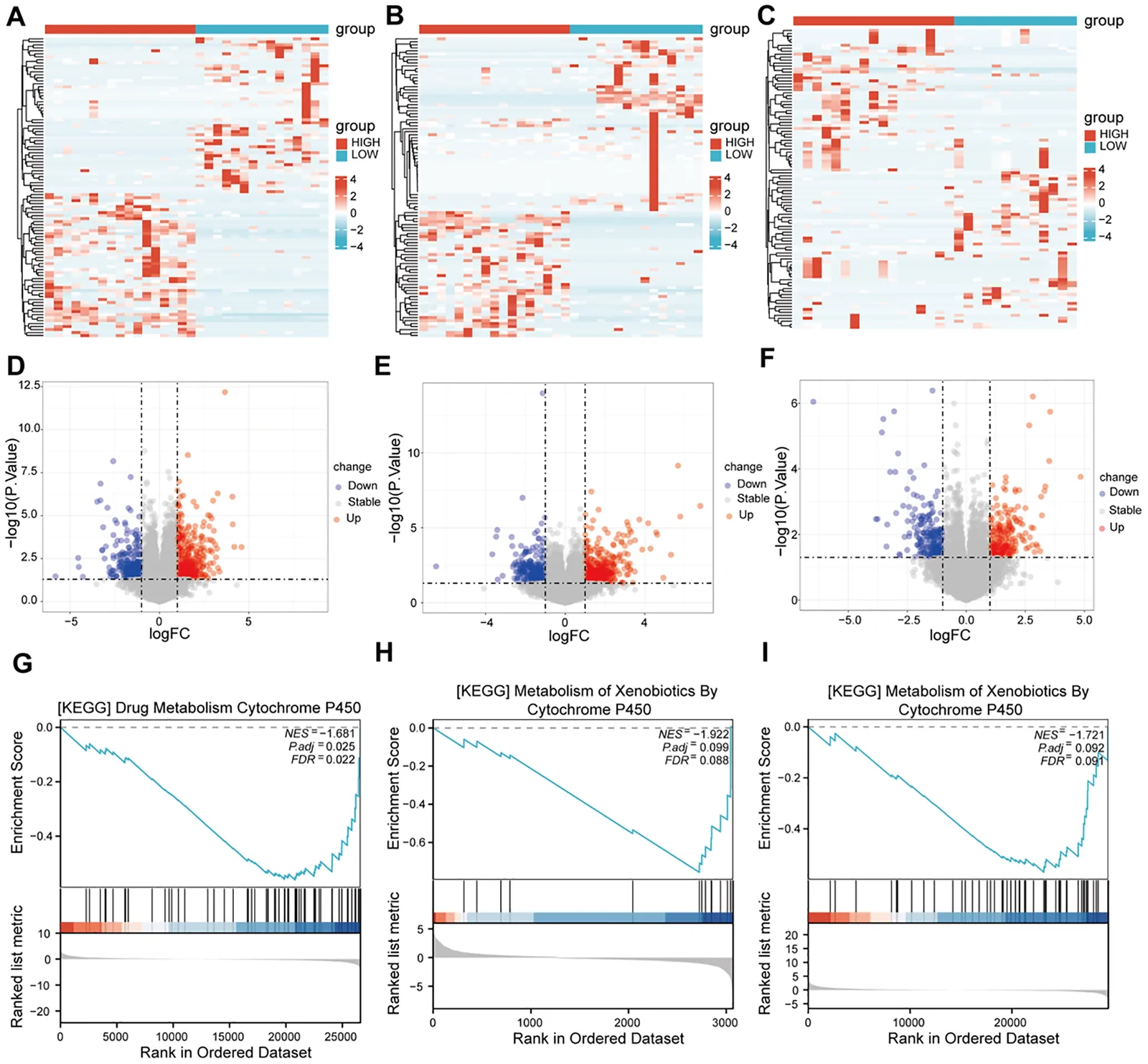
Transcriptomic profiling reveals tacrolimus metabolism-associated gene expression divergence between extreme AUC_0-t_ phenotypes in BXD strains (11 strains; n = 30 mice). **(A–C)** Tissue-specific expression heatmaps. Hierarchically clustered z-scores of significantly differentially expressed genes in: **(A)** Hepatic tissue; **(B)** Renal tissue; **(C)** The small intestine tissue. **(D-F)** Volcano plots of tissue-specific DEGs (|log2FC| > 1, *p* < 0.05): **(D)** Liver; **(E)** Kidney; **(F)** Small intestine. **(G–I)** GSEA of canonical metabolic pathways Enrichment of Hallmark gene sets (MSigDB v7.5) in: **(G)** Liver: Drug metabolism CYP450; **(H)** Kidney: Metabolism of xenobiotics by CYP450; **(I)** Intestine: Metabolism of xenobiotics by CYP450.

We divided the samples into high and low groups based on the average AUC_0-t_ value and conducted RNA sequencing on liver, kidney, small intestine tissues from 11 BXD mouse strains (**Figures 4A–C**; **Supplementary Figure 4**). For liver tissue, a total of 410 differentially expressed genes were identified, with 85 upregulated and 325 downregulated (**Figure 4D**). The GSEA results showed that the drug metabolism cytochrome P450 pathway activity was higher in the low AUC_0-t_ group compared to the high AUC_0-t_ group (**Figure 4G**). For kidney, a total of 749 differentially expressed genes were identified, with 352 upregulated and 397 downregulated (**Figure 4E**). The GSEA results showed that the metabolism of xenobiotics by cytochrome P450 pathway activity was higher in the low AUC_0-t_ group compared to the high AUC_0-t_ group (**Figure 4H**). For small intestine, a total of 443 differentially expressed genes were identified, with 274 upregulated and 169 downregulated. GO and KEGG enrichment results indicated that the differentially expressed genes were primarily enriched in terms such as metabolic pathways (**Figure 4F**). The GSEA results showed that the metabolism of xenobiotics by cytochrome P450 pathway activity was higher in the low AUC_0-t_ group compared to the high AUC_0-t_ group (**Figure 4I**).

### 3.5 Identification of candidate regulators governing tacrolimus metabolism

**Figure 5:**
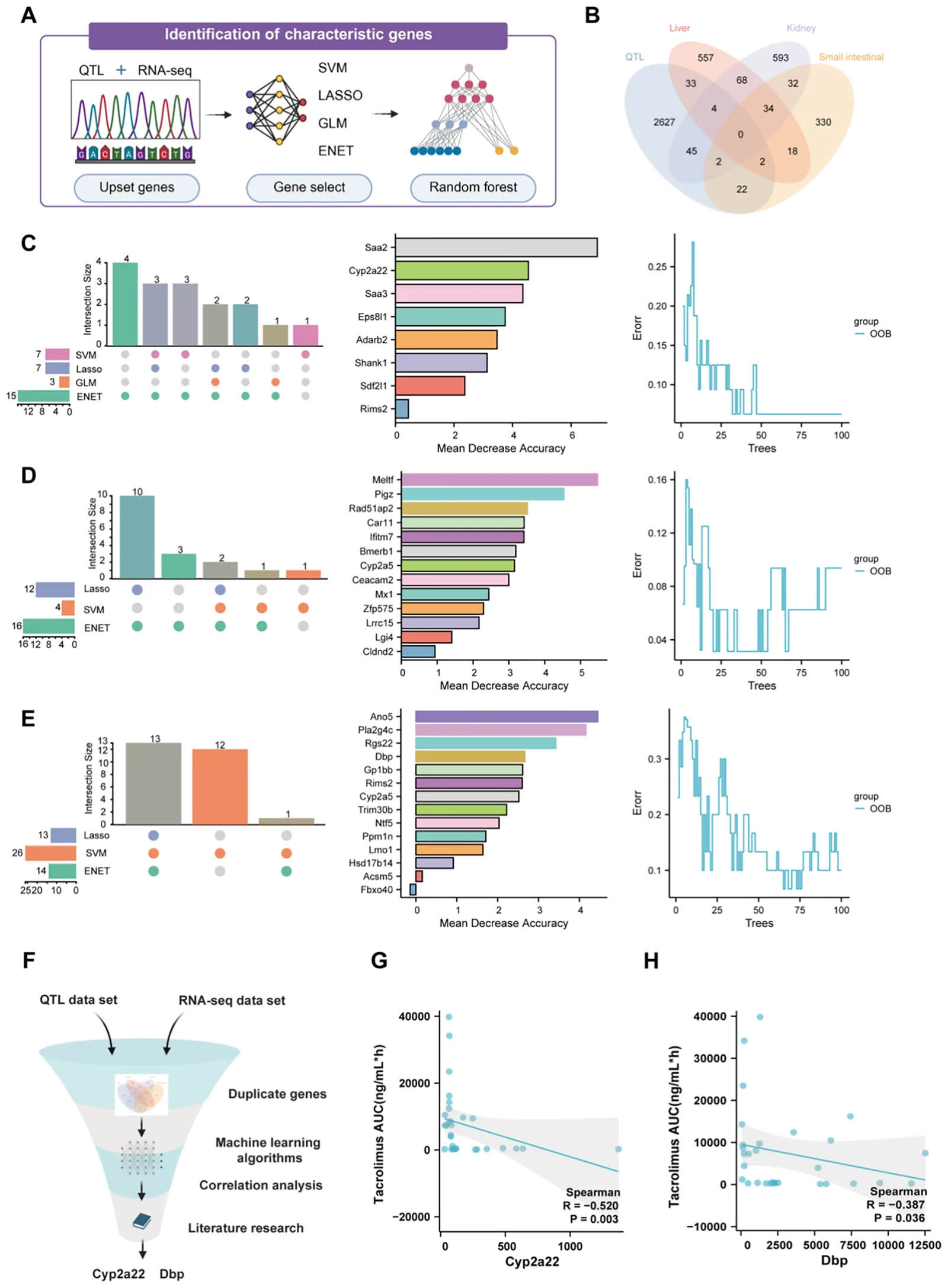
Machine learning-driven identification of candidate genetic modulators of tacrolimus metabolism. **(A)** Multi-modal feature selection workflow. **(B)** Integrative genomics intersection. Venn diagram depicting overlap between: genes within QTL support intervals and differentially expressed genes. **(C–E)** Left: Tissue-specific consensus feature genes identified by integrative machine learning. Right: Random Forest validation analysis: Variable importance rankings; Error rate convergence across 500 decision trees. **(C)** Hepatic tissue; **(D)** Renal tissue; **(E)** Intestinal tissue. **(F)** Flowchart for Screening Candidate Modulators (Cyp2a22 and Dbp) Affecting Tacrolimus Metabolic Blood Concentration via Integrated Analyses (QTL Mapping, Transcriptomics, Machine Learning, Correlation Analysis, Literature Review). **(G)** Correlation Analysis Between Cyp2a22 Expression and Tacrolimus AUC_0-t_. **(H)** Correlation Analysis Between Dbp Expression and Tacrolimus AUC_0-t_.

**Figure 6:**
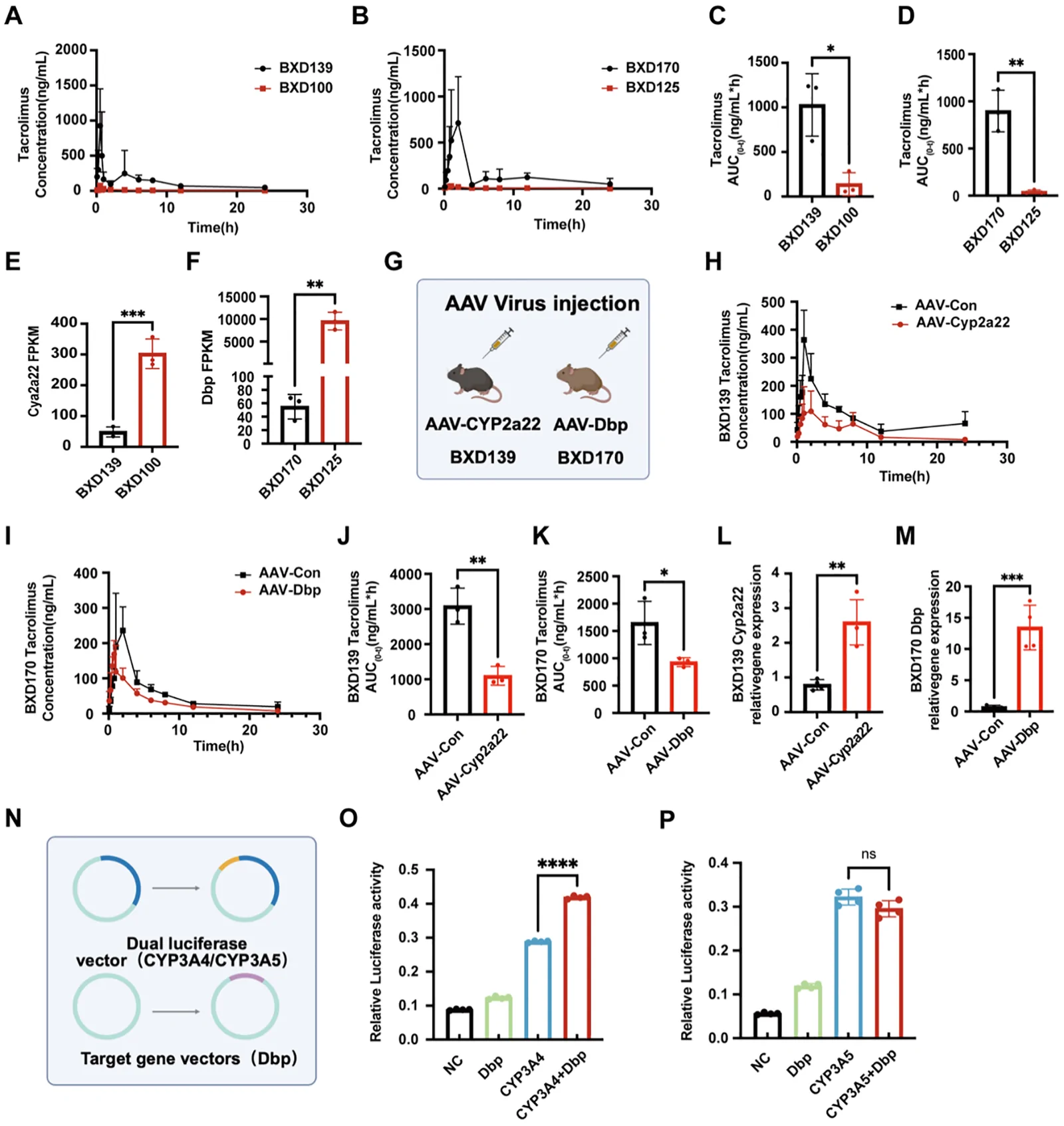
Verification of candidate genes associated with tacrolimus metabolic blood concentration. **(A)** Mean Tacrolimus Blood Concentration-Time Curves in BXD139 vs. BXD100 Mouse Strains (10 mg/kg, Oral Administration). **(B)** Mean Tacrolimus Blood Concentration-Time Curves in BXD170 vs. BXD125 Mouse Strains (10 mg/kg, Oral Administration). **(C)** Differences in Tacrolimus AUC_0-t_ Between BXD139 and BXD100 Mouse Strains. **(D)** Differences in Tacrolimus AUC_0-t_ Between BXD170 and BXD125 Mouse Strains. **(E)** Differences in Cyp2a22 FPKM Between BXD139 and BXD100 Mouse Strains. **(F)** Differences in Dbp FPKM Between BXD170 and BXD125 Mouse Strains. **(G)** Schematic Diagram of AAV-Mediated Gene Overexpression: Hepatic Cyp2a22-AAV (Tail Vein Injection for BXD139) and Intestinal Dbp-AAV (Tail Vein Injection for BXD170), each group comprised n = 3 independent biological replicates. **(H)** Mean Tacrolimus Blood Concentration-Time Curves in BXD139 (Con-AAV) vs. BXD139 (Cyp2a22-AAV) Mice (10 mg/kg, Oral Administration). **(I)** Mean Tacrolimus Blood Concentration-Time Curves in BXD170 (Con-AAV) vs. BXD170 (Dbp-AAV) Mice (10 mg/kg, Oral Administration). **(J)** Differences in Tacrolimus AUC_0-t_ Between BXD139 (Con-AAV) and BXD139 (Cyp2a22-AAV) Mice. **(K)** Differences in Tacrolimus AUC_0-t_ Between BXD170 (Con-AAV) and BXD170 (Dbp-AAV) Mice. **(L)** Differences in Cyp2a22 Relative Expression: BXD139 (Con-AAV) vs. BXD139 (Cyp2a22-AAV) Mice. **(M)** Differences in Dbp Relative Expression: BXD170 (Con-AAV) vs. BXD170 (Dbp-AAV-Injected) Mice. **(N)** Schematic of Dual-Luciferase Reporter Assay for Investigating Dbp-Mediated Regulation of CYP3A4/CYP3A5. **(O)** Dual-Luciferase Assay: DBP-Mediated Activation of the CYP3A4 Promoter. **(P)** Dual-Luciferase Assay: Dbp Fails to Activate the CYP3A5 Promoter. * p < 0.05, ** p < 0.01, *** p < 0.001, **** p < 0.0001, ns = not significant.

Next, we performed intersection analysis between the genes corresponding to the QTL loci and the differentially expressed genes identified in the high and low AUC_0-t_ groups across various tissues obtained from RNA-seq (**Figures 5A, B**). This integration yielded 33 candidate genes in the liver, 45 in the kidney, and 22 in the small intestine. The binary machine learning methods, including SVM, LASSO, GLM, and ENET, were used to screen the intersection genes. The genes that appeared in two or more of these methods were selected as key genes (**Figures 5C–E**). This refined screening yielded 8 key genes in the liver, 13 in the kidney, and 14 in the small intestine. To further identify core regulatory genes, we performed correlation analysis between the Log_2_FC of these differentially expressed genes and the top five feature importance variables. The results revealed that Cyp2a22 and Dbp not only exhibited a negative correlation with AUC_0-t_ but also showed the most substantial fold-change (Log_2_FC) among the candidate genes. After evaluating their established roles in tacrolimus metabolism within the existing literature, we ultimately pinpointed Dbp and Cyp2a22 as the candidate modulators (**Figures 6A–C**; **Supplementary Figure 5**).

### 3.6 Verification of key genes modulating tacrolimus metabolism and blood concentration

Through an integrated approach including QTL mapping, transcriptomics, machine learning, correlation analysis, and literature review, we identified Cyp2a22 and Dbp as candidate modulators of tacrolimus metabolism. BXD139 exhibited high tacrolimus blood concentrations but low Cyp2a22 expression, whereas BXD100 displayed the opposite profile. BXD170 had high tacrolimus blood concentrations but low Dbp transcript levels, with BXD125 showing the inverse pattern (**Figures 6A–F**). Accordingly, we induced hepatic overexpression of Cyp2a22 in BXD139 mice and intestinal overexpression of Dbp in BXD170 mice via AAV (**Figure 6G**; **Supplementary Table 1**). Subsequent studies demonstrated that both hepatic Cyp2a22 overexpression (BXD139-OE-Cyp2a22 mice) and intestinal Dbp overexpression (BXD170-OE-Dbp mice) significantly reduced tacrolimus AUC_0-t_ compared to their respective controls. These findings confirmed that upregulation of either gene accelerates tacrolimus disposition and decreases systemic exposure (**Figures 6H–M**). *In vitro* dual-luciferase assays further showed that DBP significantly enhanced the promoter activity of CYP3A4 (experimental group: 0.4194 ± 0.0045 vs. control group: 0.2886 ± 0.0024; *p* < 0.0001), but had no significant effect on the promoter activity of CYP3A5 (experimental group: 0.3220 ± 0.0183 vs. control group: 0.2954 ± 0.0184). These findings provided molecular evidence that DBP specifically regulated tacrolimus metabolism via CYP3A4 (**Figures 6N–P**).

### 3.7 Construction of a clinical prediction model

**Figure 7:**
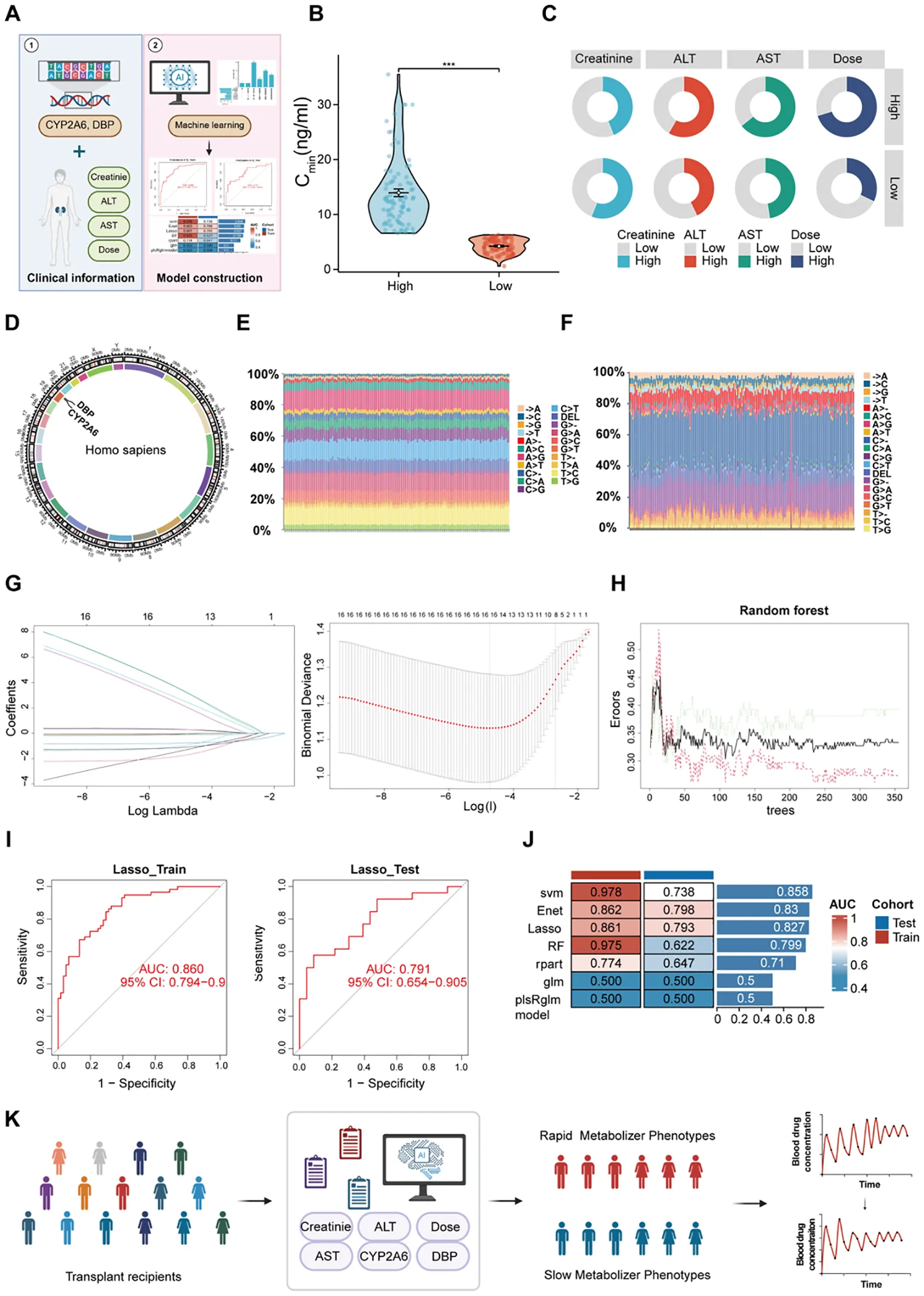
Development and validation of a tacrolimus pharmacokinetic prediction model. **(A)** Integrated predictive framework. Workflow integrating clinical variables, genotyping (DBP/CYP2A6), and machine learning algorithms. **(B)** Patient stratification 168 renal transplant recipients stratified by trough concentration Cmin: High-C_min_ group (n=84); Low-C_min_ group (n=84). **(C)** Clinical metadata distribution Donut chart profiling. **(D)** Genetic architecture Chromosomal mapping of DBP and CYP2A6 on GRCh38 assembly. **(E, F)** Mutational landscapes of CYP2A6 and DBP in renal transplant recipients. **(G)** LASSO coefficient trajectory Feature shrinkage profile. **(H)** Random Forest optimization. Error rate convergence at 500 trees. **(I)** Model discrimination ROC curves: Training AUROC = 0.861 (95% CI: 0.794-0.918), Test AUROC = 0.791 (0.654-0.905). **(J)** Multi-algorithm performance. Heatmap comparing AUROC metrics. **(K)** Mechanistic paradigm. *** p < 0.001.

We retrospectively collected whole-blood samples from 168 renal transplant recipients receiving tacrolimus-based immunosuppression. For cross-species comparison, mouse gene symbols were converted to their human orthologs, with Dbp corresponding to DBP and Cyp2a22 linked to CYP2A6 on the basis of orthology annotations from NCBI Gene (Gene ID: 233005), MGI (MGI:3648316), and the Alliance of Genome Resources. These resources identify CYP2A6 as one of three reported human orthologs of Cyp2a22, together with CYP2A13 and CYP2A7. Among these candidates, CYP2A6 was prioritized because of its established role in xenobiotic metabolism and prior pharmacogenomic evidence suggesting an association between CYP2A6 variants and tacrolimus exposure (**Figure 7A**) (30).

Using the median C_min_ as an operational cut-off for statistical stratification, patients were stratified into high- (n = 84) and low-exposure (n = 84) cohorts. This grouping strategy was used for binary model construction and does not directly correspond to clinically defined therapeutic or toxic tacrolimus concentration ranges. The high-exposure group had a median Cmin of 12.5 ng/mL (IQR, 9.4-16.0), whereas the low-exposure group had a median Cmin of 4.3 ng/mL (IQR, 3.2-5.3) (**Figures 7B, C**; **Table 1**). The available baseline demographic, dosing, and biochemical characteristics of the clinical cohort are summarized in **Table 1**. Both DBP and CYP2A6 are located on chromosome 19 (**Figure 7D**). The full-length genes of DBP and CYP2A6 were sequenced across all collected samples, followed by PLINK association analysis, ultimately revealing variant sites between the high- and low-exposure groups (**Figures 7E, F**; **Supplementary Figure 5**). The machine-learning algorithms were employed to distill the key feature parameters differentiating the high- and low-exposure cohorts (**Supplementary Figure 5**). Subsequently, multiple predictive models were constructed and evaluated using receiver-operating characteristic analysis and area under the curve (AUROC) values, with the LASSO model showing the most favorable overall performance (**Figures 7G–J**; **Supplementary Figure 6**).

**Table 1:** Baseline characteristics of the 168 renal transplant recipients.

Variable	Overall (n = 168)	Low-concentration group (n = 84)	High-concentration group (n = 84)	P value
No. of recipients	168	84	84	
Age (years)	46.5 (40.0-55.0)	45.0 (37.8-53.0)	47.5 (41.0-55.0)	0.078
Male sex, n (%)	123 (73.2)	64 (76.2)	59 (70.2)	0.384
Female sex, n (%)	45 (26.8)	20 (23.8)	25 (29.8)	
Serum creatinine, (μmol/L)	844.0 (685.8-1090.5)	846.6 (685.8-1131.1)	826.9 (690.6-1073.6)	0.606
ALT (U/L)	14.0 (10.0-20.0)	13.0 (10.0-20.0)	14.0 (10.0-19.8)	0.541
AST (U/L)	15.0 (12.0-19.0)	15.0 (11.8-19.0)	14.5 (12.0-19.8)	0.702
Initial tacrolimus dose (mg)	3.0 (2.0-3.5)	2.0 (2.0-3.0)	3.0 (2.5-4.0)	<0.001
Initial tacrolimus trough concentration (ng/mL)	6.4 (4.4-12.5)	4.3 (3.2-5.3)	12.5 (9.4-16.0)	<0.001

Continuous variables are presented as median (interquartile range, IQR), and categorical variables are presented as n (%). P values compare the low- and high-concentration groups and were calculated using the Wilcoxon rank-sum test for continuous variables and the chi-square test for categorical variables. ALT and AST were available for 134 recipients overall, including 60 in the low-concentration group and 74 in the high-concentration group; missing values were excluded from variable-specific summaries.

To further assess model robustness and quantify the incremental contribution of genetic variables, we implemented 5-fold nested cross-validation, in which feature selection and model tuning were performed exclusively within each outer training fold, thereby preventing information leakage from the outer test partitions (**Supplementary Figure 7**). The full model incorporating DBP and CYP2A6 genetic variants alongside clinical variables achieved a mean nested CV AUROC of 0.771 ± 0.081. A parallel model using clinical variables alone (creatinine, ALT, AST, and tacrolimus dose) yielded a mean nested CV AUROC of 0.725 ± 0.058, demonstrating an incremental ΔAUROC of 0.045 attributable to the genetic features. Calibration analysis based on out-of-fold predicted probabilities from the nested cross-validation framework showed acceptable overall agreement between predicted and observed probabilities in this exploratory setting, although calibration slopes below 1 suggested some degree of overfitting (**Supplementary Figure 7**).

## 4 Discussion

Tacrolimus serves as a cornerstone immunosuppressive agent in solid organ transplantation, including kidney, liver, and lung grafts (31, 32). Despite its irreplaceable role, achieving precision in tacrolimus dosing remains a persistent clinical challenge (5). Previous efforts have largely relied on single-strain rodent models, which fail to capture the polygenic complexity underlying human pharmacokinetic variation (33). Conversely, clinical studies in transplant recipients are constrained by an inability to access tissue-level drug distribution and by the practical difficulties of longitudinal sampling in heterogeneous human populations. These methodological gaps underscore the urgent need for a translational model that integrates genetic diversity with the experimental controllability required to dissect drug disposition mechanisms (34, 35). The BXD recombinant inbred mouse platform constitutes a genetically diverse reference population that is well suited for mechanistic discovery under controlled experimental conditions and may help inform future translational studies.

In this study, we have employed the BXD platform to provide a comprehensive systems-genetic dissection of tacrolimus pharmacokinetics, integrating high-density genomic data with multi-omics profiling across a large panel of strains (36). Across the 46 BXD strains, significant inter-strain variations in tacrolimus metabolism were observed, likely driven by genetic influences. Leveraging these pharmacokinetic profiles, we identified 19 genomic loci associated with tacrolimus disposition through QTL mapping. This establishes that tacrolimus metabolism is polygenically regulated rather than controlled by a single gene, operating through multiple genetic pathways. To mechanistically dissect this complexity, we performed transcriptomic profiling on strains exhibiting the most extreme metabolic divergence, integrating QTL positional data to prioritize causal candidate genes.

Integrating *in vivo* pharmacokinetic studies with in silico analyses, we identified Dbp and Cyp2a22 as candidate genetic modulators associated with tacrolimus blood concentrations in mice. These findings were further supported through adeno-associated virus (AAV)-mediated manipulation in BXD mice.

Tacrolimus undergoes extensive metabolism by CYP450 enzymes, primarily in the liver and intestine. CYP3A5 is currently the best-validated pharmacogenetic marker for tacrolimus initial dose selection and has recognized clinical relevance in implementation guidelines. (11, 15, 37). In this context, our findings are not intended to challenge the central role of CYP3A5, but rather to suggest that additional modulators may contribute to the residual interindividual variability in tacrolimus exposure that is not fully explained by CYP3A5 alone. This perspective is consistent with the broader clinical experience that genotype-guided tacrolimus dosing based on CYP3A5 can improve early exposure attainment, yet does not fully resolve the complexity of tacrolimus pharmacokinetics in transplant recipients.

Within this framework, our systems-genetics analysis identified Cyp2a22 and Dbp as candidate genetic modulators associated with blood tacrolimus concentrations. Previous reports have implicated Cyp2a22 in bile acid metabolism and have documented its mutational involvement in bladder cancer (38, 39). Of translational relevance, CYP2A6 represents one of the closest human orthologs of murine Cyp2a22 (NCBI Gene ID: 233005; MGI:3648316) (39). From a translational perspective, CYP2A6 is a well-characterized human cytochrome P450 enzyme involved in the metabolism of multiple xenobiotics and clinically relevant drugs. (40). It is the principal enzyme responsible for the oxidation of nicotine, influencing addiction propensity and smoking behavior (41). Beyond this, CYP2A6 actively metabolizes several clinically important drugs, including the chemotherapeutic agent tegafur, the muscle relaxant halothane, and various other medications (42, 43). Furthermore, it plays a critical role in the bioactivation of tobacco-specific procarcinogens, such as nitrosamines, linking its activity to cancer risk (44). Notably, a pharmacogenomic study utilizing machine learning identified rs1137115 in CYP2A6 as a candidate single-nucleotide polymorphism (SNP) affecting tacrolimus exposure in a Korean male cohort (30). However, the translation of these findings to humans requires caution. Although murine Cyp2a22 and human CYP2A6 share reported orthologous relationships, orthology does not necessarily imply equivalent substrate specificity, regulatory behavior, or clinical importance. D site-binding protein (DBP), is a core circadian transcription factor (45). It specifically recognizes and binds to a specific DNA sequence motif, known as the D-box element, located within the promoter or enhancer regions of its target genes (46). Notably, the regulatory regions of many genes encoding hepatic drug-metabolizing enzymes contain these conserved D-box elements (47, 48). Studies indicate that in mice, Dbp acts as a transcription factor regulating the expression of Cyp3a11, while in human-derived cells, DBP transcriptionally regulates CYP3A4 (49, 50). Notably, CYP3A4 and CYP3A5 are the primary and secondary human orthologs of murine Cyp3a11, respectively (51, 52). Through dual-luciferase reporter assays, our results further confirm that DBP exerts transcriptional regulatory effects on CYP3A4 (53). Interestingly, tacrolimus blood concentrations have been shown to exhibit significant circadian oscillations, a phenomenon under the control of the molecular circadian clock (54, 55).

More broadly, pharmacogenomic strategies for tacrolimus in human populations have already expanded beyond single-gene paradigms to include pharmacogenomic arrays, targeted genotyping panels, sequencing-based approaches, and multi-gene predictive frameworks. Within this broader landscape, the value of the BXD platform in our study lies not in substituting for human pharmacogenomic validation, but in providing a controlled systems-genetics framework for mechanistic discovery and hypothesis generation. In this sense, the murine platform and human pharmacogenomic studies should be viewed as complementary rather than competing approaches.

The exploratory clinical modeling component of the study provides preliminary support for this translational direction. In the present study, we established a novel predictive framework based on machine learning algorithms. By integrating candidate genetic signals with selected clinical variables, the combined model showed improved discrimination relative to the clinical-only model under leakage-controlled nested cross-validation. These findings suggest that candidate genetic information may contribute to future efforts aimed at refining individualized tacrolimus exposure prediction and informing initial dosing strategies, although further validation in larger and clinically diverse cohorts will be required before clinical implementation.

Nevertheless, this study has several limitations. The clinical prediction model was developed in a relatively small, single-center retrospective cohort, which limits generalizability and increases the risk of overfitting. In addition, several clinically relevant covariates, including concomitant medications and other potential confounders, were not systematically available in the current dataset, and residual confounding therefore cannot be excluded. The exposure groups were defined using the cohort median C_min_ for statistical stratification rather than clinically established therapeutic thresholds, and sensitivity analyses based on clinically defined therapeutic ranges could not be performed. Moreover, CYP3A5, the most established pharmacogenetic marker for tacrolimus initial dose adjustment, was not systematically incorporated into the current clinical modeling framework, preventing direct assessment of whether DBP/CYP2A6 adds predictive value beyond established pharmacogenetic and clinical predictors. Although we implemented leakage-controlled nested cross-validation and further assessed calibration using out-of-fold predicted probabilities, the calibration slopes below 1 suggest some degree of overfitting, and no independent external validation cohort was available. Finally, the translational interpretation of DBP/CYP2A6 should remain cautious, because the orthologous relationship between murine Cyp2a22 and human CYP2A6 does not by itself establish equivalent biological function, and the current human cohort findings remain exploratory. Taken together, these limitations indicate that the present findings should be interpreted as mechanistic and hypothesis-generating rather than as definitive evidence for clinical implementation.

In summary, our study supports the value of the BXD mouse platform as a systems-genetics framework for investigating tacrolimus pharmacokinetic variability under controlled experimental conditions. By integrating genetic mapping, transcriptomic analysis, and functional validation, we identified Dbp and Cyp2a22 as candidate modulators of tacrolimus disposition in the murine model and generated preliminary translational hypotheses involving DBP/CYP2A6 in humans. These findings provide an experimental and computational framework for future studies aimed at validating candidate modulators and refining individualized tacrolimus dosing strategies in clinically diverse human cohorts.

## 5 Conclusion

This study uses the BXD mouse platform to investigate genetic contributors to tacrolimus pharmacokinetic variability. By characterizing pharmacokinetics across 46 strains, we identified 19 genomic loci and supported DBP/CYP2A6 as candidate modulators requiring further validation. A clinical prediction model integrating these candidate signals and selected clinical variables in 168 renal transplant recipients showed encouraging discriminatory performance, although the clinical findings remain exploratory. Overall, this work provides a mechanistic and computational framework that may inform future studies on individualized tacrolimus dosing.

## Data Availability

Datasets are available on request: The raw data supporting the conclusions of this article will be made available by the authors, without undue reservation.
