# Supplementary material for: Advancing precision tacrolimus therapy: a systems genetics dissection in BXD platform
Source: Front Immunol. 2026 Aug 26;17:1903968. doi: 10.3389/fimmu.2026.1903968 (PMC13553470; doi:10.3389/fimmu.2026.1903968)
Supplement: Supplementary file 1 [file DataSheet1.pdf]

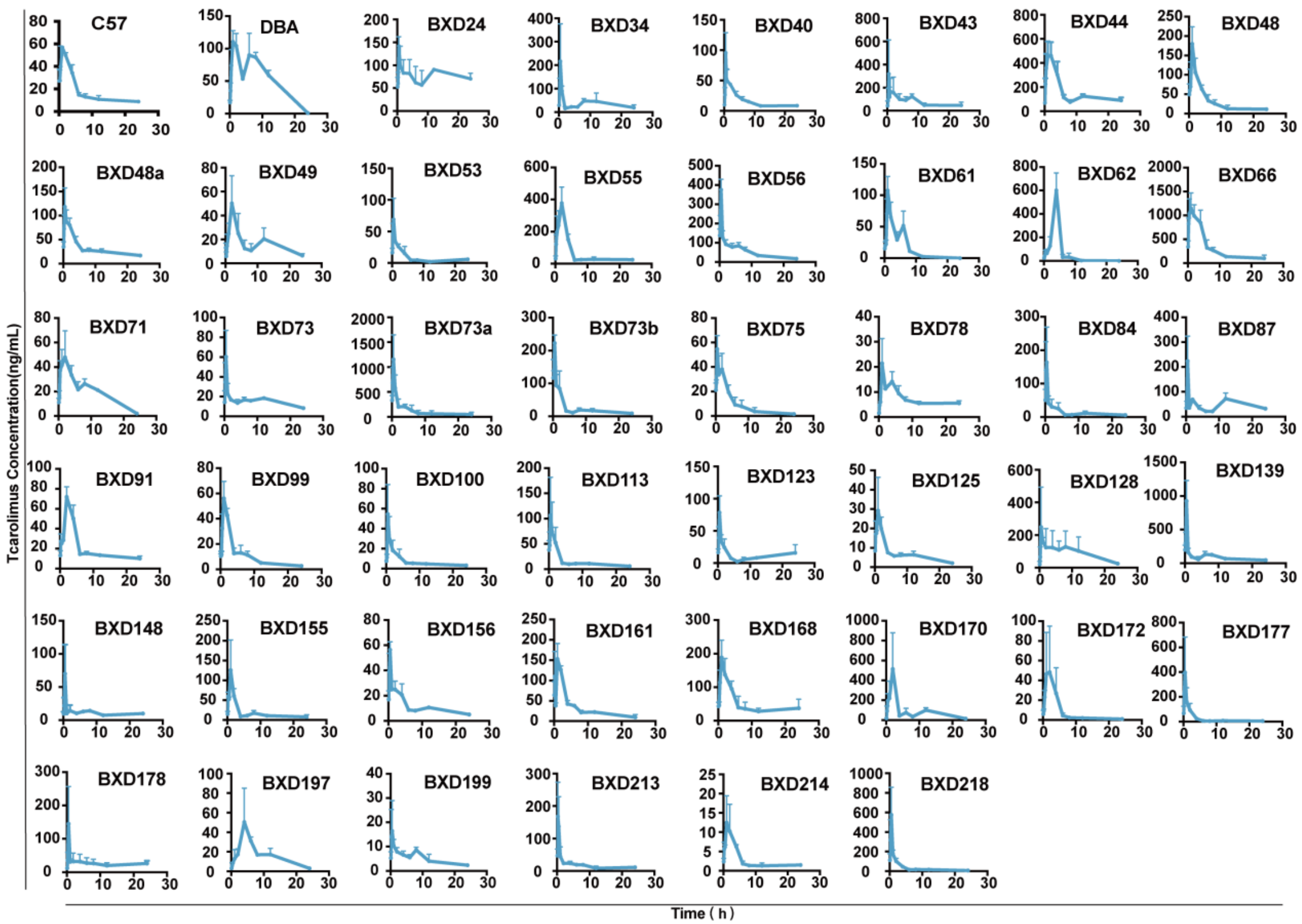

Figure S1. Blood Drug Concentration-Time Curves of Tacrolimus in 46 Strains of BXD Mice. (n = 46 strains).

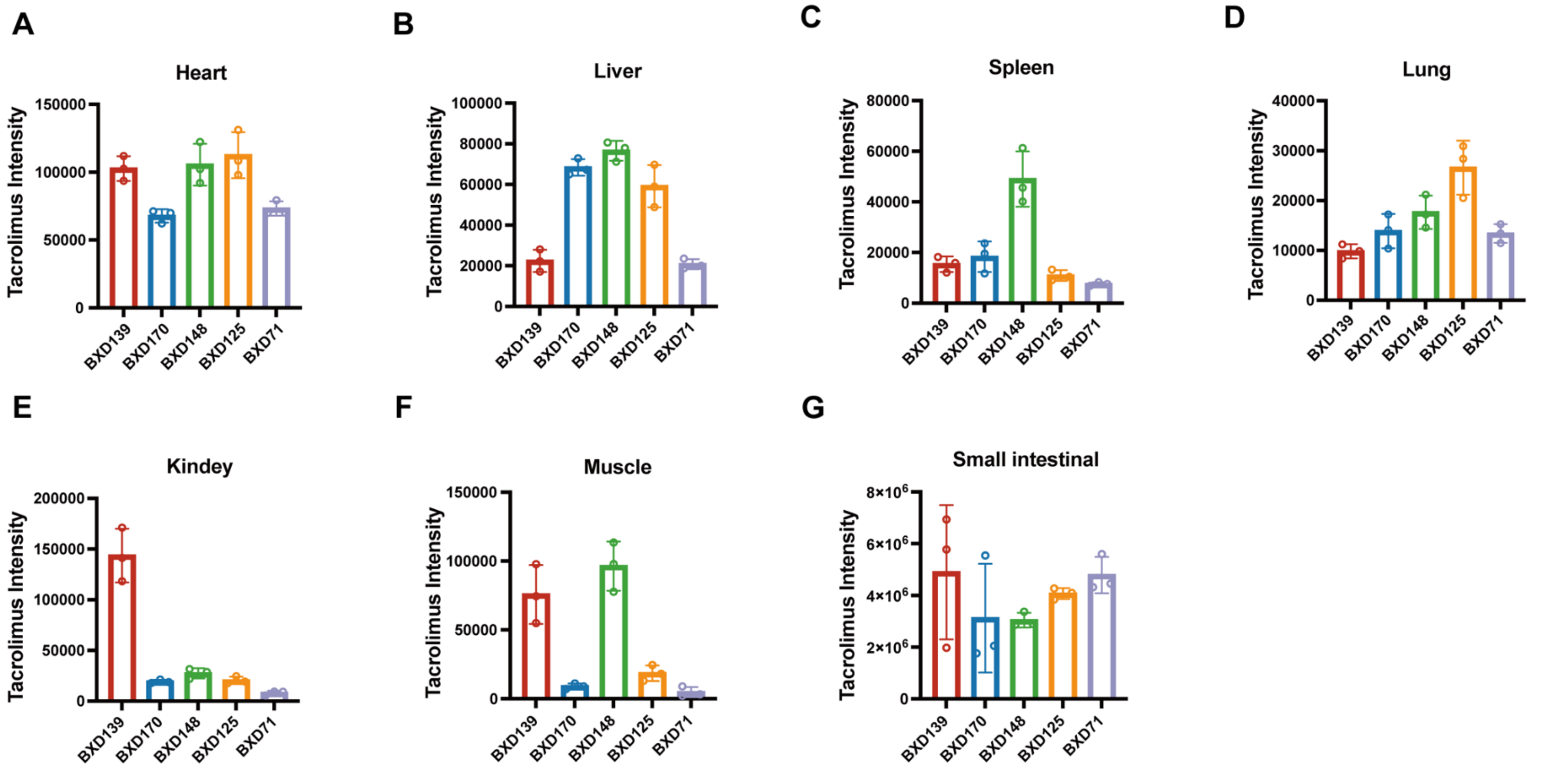

Figure S2. Spatial Metabolic Imaging of Tacrolimus in Various Organs of Different BXD Mouse Strains (BXD139, BXD170, BXD148, BXD125 and BXD71). **(A-G)**. The tacrolimus intensity of strains in the hearts, livers, spleens, lungs, kidneys, muscles, and small intestines （n = 3）.

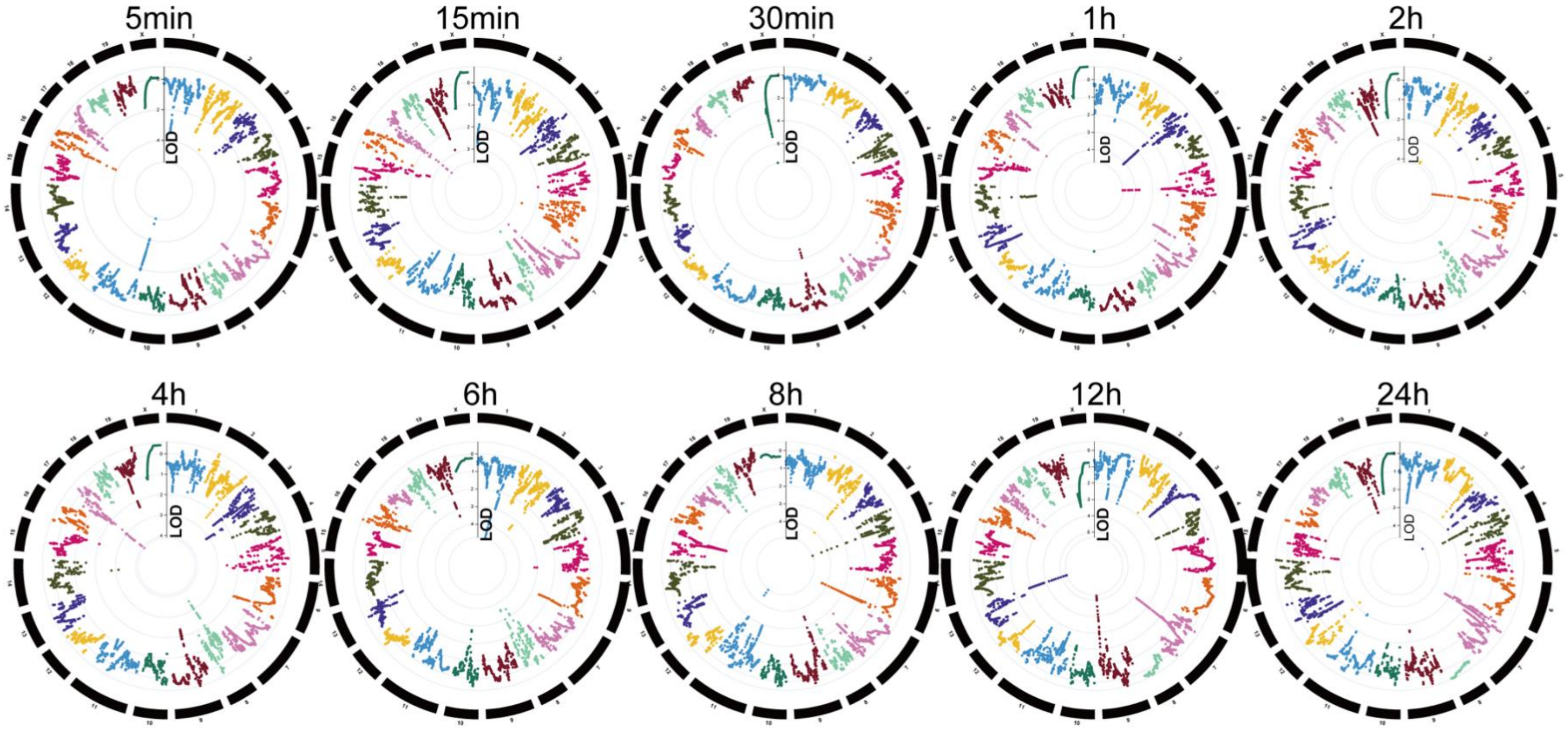

Figure S3. QTL mapping of tacrolimus blood concentrations at different time points after administration in BXD mice. (n = 46 strains; Haley-Knott regression with 10,000 permutations).

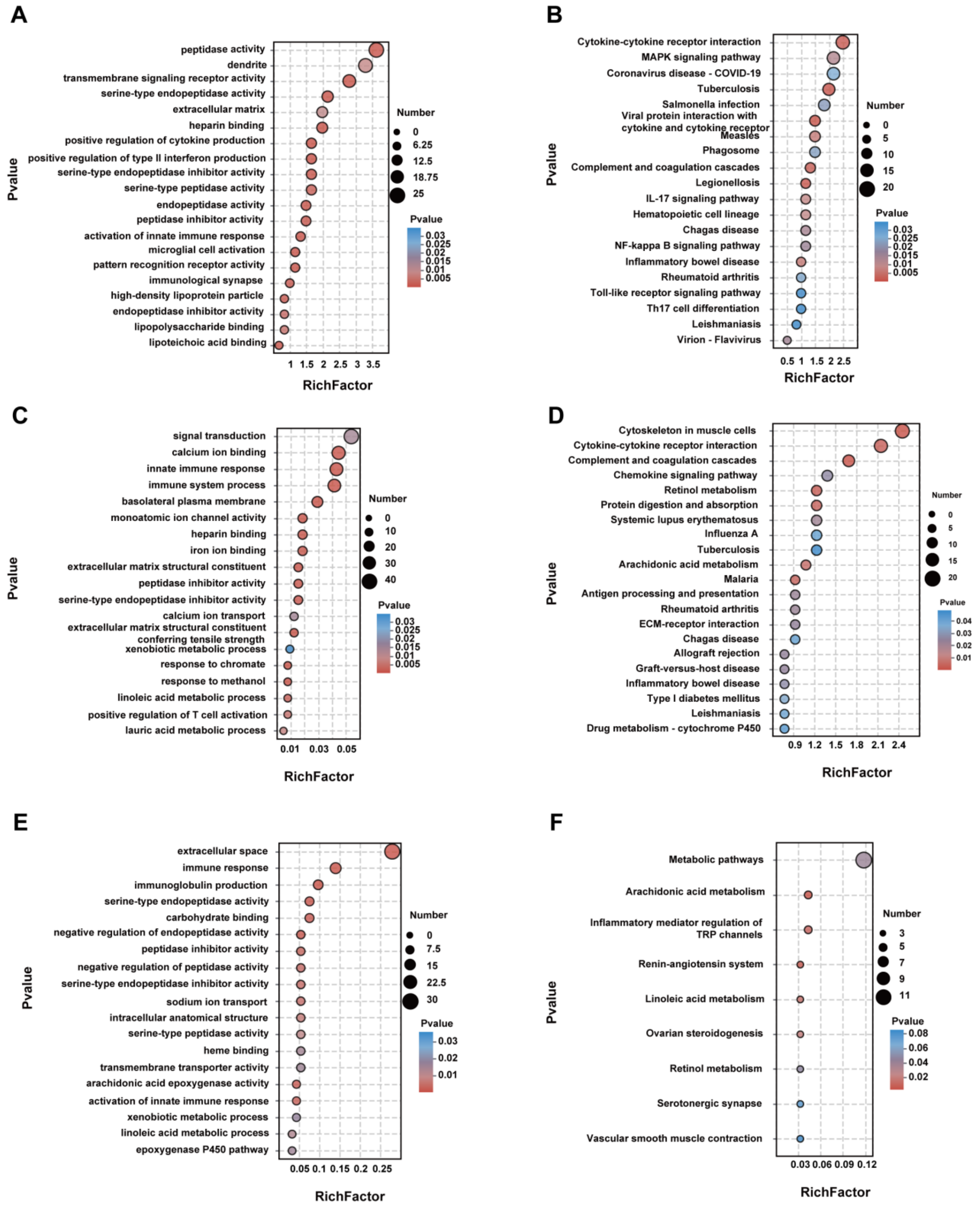

Figure S4. Enrichment analysis of DEGs in BXD strains exhibiting extreme AUC differences. (A-B) Bubble plots showing the GO and KEGG pathway enrichment analyses of DEGs in liver tissue. (C-D) GO and KEGG enrichment analyses of DEGs in kidney tissue. (E-F) GO and KEGG enrichment analyses of DEGs in small intestinal tissue.

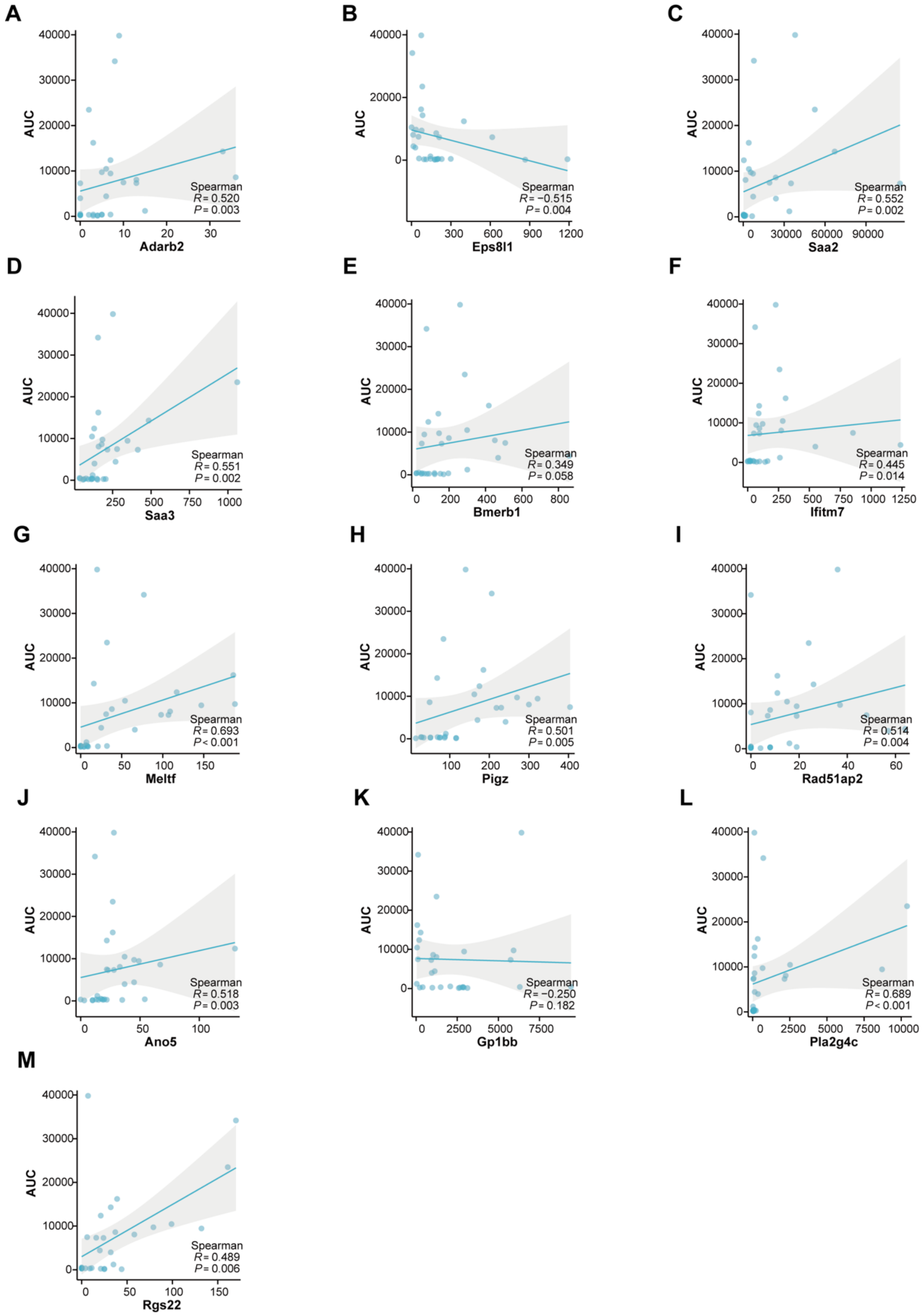

Figure S5. Correlation Analysis Between Key Genes Expression and Tacrolimus $AUC_{0\text{-}t}$. (A) Adarb2. (B) Eps8I1. (C) Saa2. (D) Saa3. (E) Bmerb1. (F) Ifitm7. (G) MeItf. (H) Pigz. (I) Rad51ap2. (J) Ano5. (K) Gp1bb. (L) Pla2g4c. (M) Rgs22.

A

Gene name	Chr	HGVS nomenclature	p	Genomic positions
DBP	chr19	NM_001352.5:c.*160_*161del	0.008133	49133933-49133934
DBP	chr19	NM_001352.5:c.551-102del	0.01831	49137016
DBP	chr19	NM_001352.5:c.562del:p.Arg188GlyfsTer15	0.03179	49136901
DBP	chr19	NM_001352.5:c.669del:p.Phe223LeufsTer19	0.03429	49136796
DBP	chr19	NM_001352.5:c.139+290del	0.04118	49139825
DBP	chr19	NM_001352.5:c.481_482del:p.Ser161ProfsTer45	0.04422	49138905-49138906
CYP2A6	chr19	NM_000762.6:c.*95del	0.01345	41349610
CYP2A6	chr19	NM_000762.6:c.813del:p.Leu272SerfsTer17	0.02427	41352800
CYP2A6	chr19	NM_000762.6:c.344-452A>T	0.02427	41355120
CYP2A6	chr19	NM_000762.6:c.344-283_344-282insTCTCTCTCTCT	0.03187	41354950-41354951
CYP2A6	chr19	NM_000762.6:c.1162-71del	0.04422	41350752
CYP2A6	chr19	NM_000762.6:c.832-18del	0.04422	41352022
CYP2A6	chr19	NM_000762.6:c.832-277_832-266del	0.04722	41352268-41352279

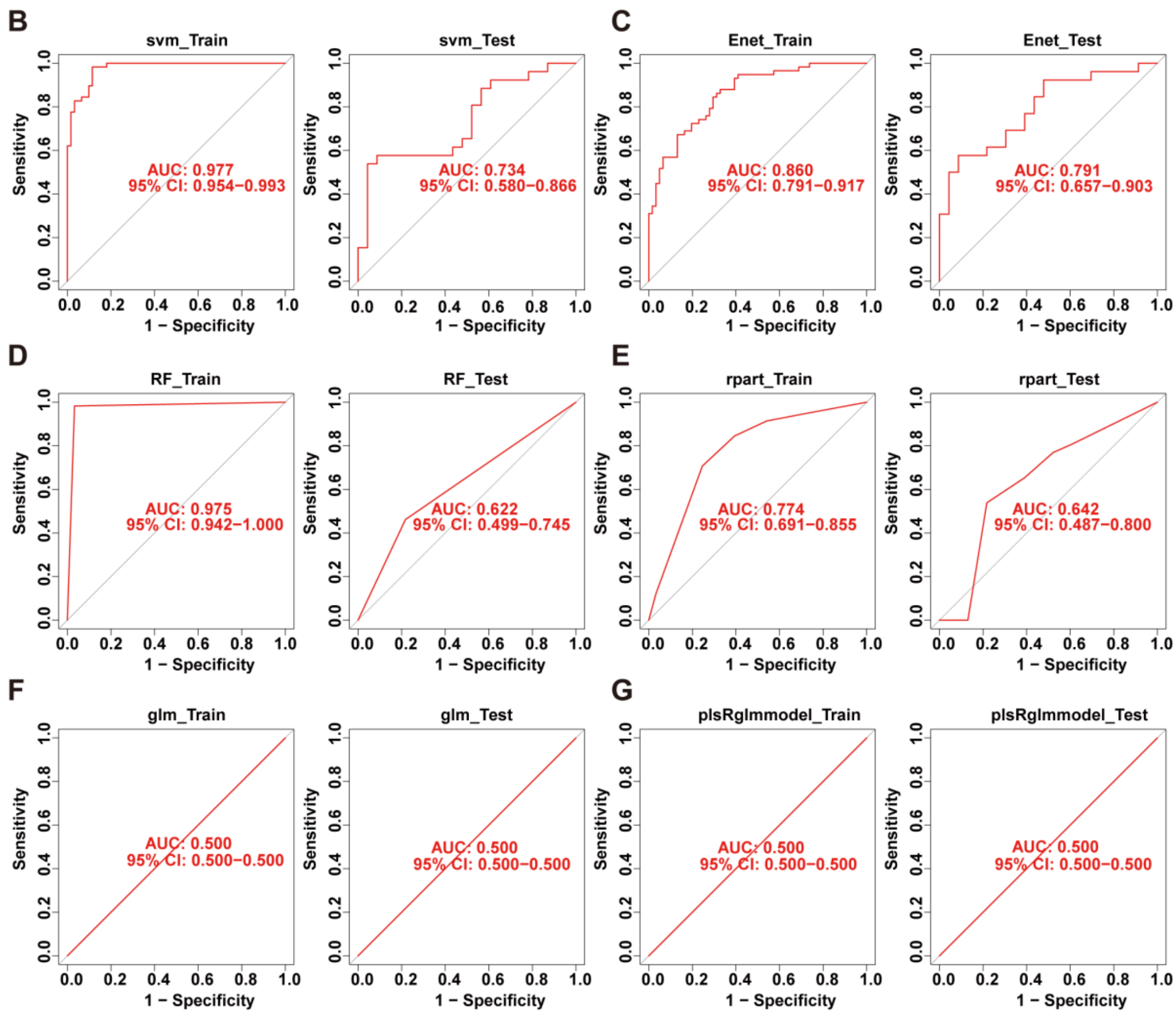

Figure S6. Construction and evaluation of a tacrolimus concentration prediction model. (A) Genetic variants of *CYP2A6* and *DBP* identified within the patient cohort. (B-G) ROC curves demonstrating the performance of various machine learning algorithms in predicting tacrolimus concentration strata.

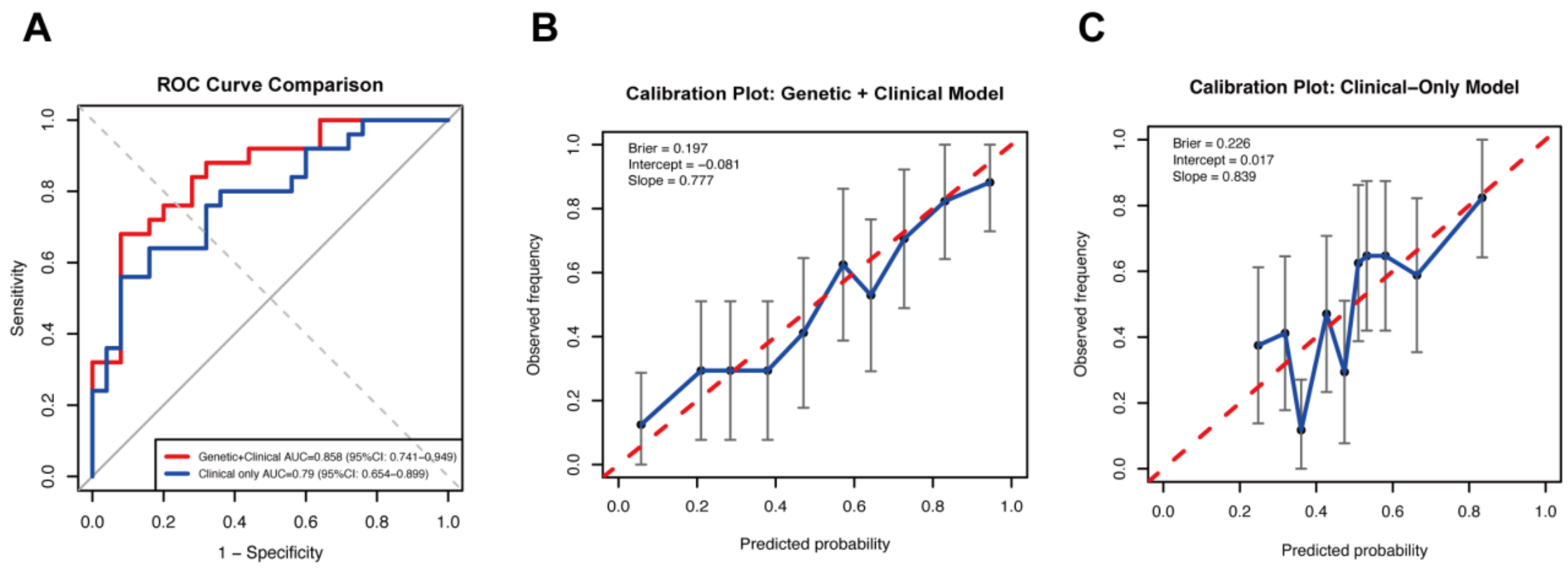

Figure S7. Performance evaluation of the clinical prediction models. (A) ROC comparison between the combined genetic-clinical model and the clinical-only model. (B) Calibration plot for the combined genetic-clinical model based on out-of-fold predicted probabilities from 5-fold nested cross-validation. (C) Calibration plot for the clinical-only model based on out-of-fold predicted probabilities from 5-fold nested cross-validation.

Table S1 Primer sequences for qRT-PCR

Gene	Forward primers（5’-3’）	Reverse primer（5’-3’）
CYP2a22	GGGGACCGCTTCAACTACG	GTCCAGGGTACTGTGGTTGTG
Dbp	TGGAGAAGACTCAATGAGGCA	GCAATACGGACTTGCTCACAGA

Table S2 QTL loci identified across five tacrolimus pharmacokinetic traits in BXD

Trait	Chr	Position (cM)	LOD Score	Permutation Threshold ($p<0.05$)	Permutation Threshold ($p<0.63$)	Classification
AUC_{0-t}	2	30.3	5.89	4.78	2.64	Genome-wide significant
AUC_{0-t}	7	18.2	3.88	4.78	2.64	Suggestive
AUC_{0-t}	13	84.9	3.73	4.78	2.64	Suggestive
AUC_{0-t}	16	2	4.11	4.78	2.64	Suggestive
AUC_{0-t}	18	97.5	6.61	4.78	2.64	Genome-wide significant
C_{max}	2	30.3	3.57	4.6	2.61	Suggestive
C_{max}	4	26.7	3.37	4.6	2.61	Suggestive
C_{max}	7	18.7	4.62	4.6	2.61	Genome-wide significant
C_{max}	8	93.8	4.05	4.6	2.61	Suggestive
C_{max}	9	126.1	4.18	4.6	2.61	Suggestive
C_{max}	12	144.9	3.82	4.6	2.61	Suggestive
C_{max}	13	85	4.01	4.6	2.61	Suggestive
C_{max}	14	69.1	3.26	4.6	2.61	Suggestive
C_{max}	15	0.1	3.13	4.6	2.61	Suggestive
C_{max}	16	3	3.5	4.6	2.61	Suggestive
C_{max}	18	45.9	5.61	4.6	2.61	Genome-wide significant
T_{max}	18	94.2	3.61	4.88	2.66	Suggestive
$T_{1/2}$	6	211	4.12	5.36	2.71	Suggestive
$T_{1/2}$	18	21	3.1	5.36	2.71	Suggestive
